# Assessment of Survival of Pediatric Patients With Hepatoblastoma Who Received Chemotherapy Following Liver Transplant or Liver Resection

**DOI:** 10.1001/jamanetworkopen.2019.12676

**Published:** 2019-10-04

**Authors:** Jincheng Feng, Ying He, Lai Wei, Dong Chen, Huifang Yang, Rumeng Tan, Zhishui Chen

**Affiliations:** 1 Institute of Organ Transplantation, Tongji Hospital, Tongji Medical College, Huazhong University of Science and Technology, Wuhan, China; 2 Department of Surgery, University of Heidelberg, Heidelberg, Germany; 3 Department of Liver Surgery, Liver Transplantation Center, West China Hospital of Sichuan University, Chengdu, Sichuan Province, China

## Abstract

**Importance:**

The incidence of hepatoblastoma is increasing, and liver transplant (LT) provides a potential cure for pediatric patients with unresectable hepatoblastoma; however, the use of LT for hepatoblastoma has not been examined in a modern cohort. Moreover, data are lacking on the association between the type of surgical management received and overall risk of death among pediatric patients with hepatoblastoma.

**Objectives:**

To examine the receipt of LT among pediatric patients with hepatoblastoma and to assess overall survival of pediatric patients with hepatoblastoma who were treated with chemotherapy after LT or liver resection (LR) using data from a national cancer registry.

**Design, Setting, and Participants:**

This cohort study used data for 443 pediatric patients with histologically confirmed hepatoblastoma who received chemotherapy and surgical therapies, as documented in the Surveillance, Epidemiology, and End Results database of the National Cancer Institute, from 2004 to 2016, with follow-up through December 31, 2018. Multivariable logistic regression was used to determine factors associated with the use of LT. Cox proportional hazards models were used to assess factors associated with overall survival. Data analysis was performed from April 18, 2019, to July 25, 2019.

**Main Outcomes and Measures:**

Overall survival.

**Results:**

Among 443 patients receiving chemotherapy (mean [SD] age, 1.8 [2.6] years; 167 [37.7%] female), 350 (79%) underwent LR and 93 (21%) underwent LT. Multivariable analysis showed that patients with multiple lesions were more likely to undergo LT than LR (31% vs 13%; *P* < .001) and that patients with higher stage tumors were more likely to undergo LT than LR (local disease, 20% vs 58%; regional disease, 58% vs 24%; distant disease, 22% vs 18%; *P* < .001). There was a statistically significant 19% increase in the receipt of LT from 8% in 1998 to 27% 2016 (trend test, *P* = .02). Overall survival at 10 years was not significantly different for the 2 surgical management strategies (87.2% [95% CI, 78.3%-97.1%] for patients undergoing LT vs 87.8% [95% CI, 83.5%-92.4%] for those undergoing LR; *P* = .92). The overall risk of death was not significantly different for LT compared with LR (hazard ratio, 0.716; 95% CI, 0.309-1.657; *P* = .44).

**Conclusions and Relevance:**

The use of LT for the management of hepatoblastoma has increased significantly over time. Among pediatric patients with hepatoblastoma receiving chemotherapy, LT was not associated with improved overall survival compared with LR. There was no significant different between treatments with regard to the outcome variable, but this finding cannot be interpreted as indicating equivalence or lack of superiority.

## Key Points

**Question:**

Is there a survival benefit associated with liver transplant vs resection in pediatric patients with hepatoblastoma receiving chemotherapy?

**Findings:**

In this cohort study of 443 pediatric patients with hepatoblastoma, 93 were treated with liver transplant, with increasing proportional use from 1998 to 2016. Compared with patients undergoing liver resection, liver transplant was not associated with improved overall survival among pediatric patients receiving chemotherapy.

**Meaning:**

This study suggests that liver transplant is not significantly associated with improved overall survival compared with liver resection combined with current chemotherapy regimens in pediatric patients with hepatoblastoma.

## Teaser

This cohort study uses Surveillance, Epidemiology, and End Results data to examine whether there is a survival benefit associated with liver transplant vs resection in pediatric patients with hepatoblastoma receiving chemotherapy.

## Introduction

Hepatoblastoma is the most common primary liver malignant neoplasm in pediatric patients, with an annual incidence of 1.5 patients per million population.^1^ With advances in surgical management and administration of chemotherapy, the 5-year overall survival (OS) rate for pediatric patients with hepatoblastoma has increased to greater than 80%.^2,3,4^ Surgical resection is the primary treatment for pediatric patients with hepatoblastoma; however, approximately 60% of patients present with unresectable hepatoblastoma at the time of diagnosis.^5,6^ The introduction of chemotherapy has greatly improved resectability for patients with stage III and IV hepatoblastoma.^7^ A 5-year survival rate of 88% has been reported for patients with posttreatment extent of disease stage III and IV hepatoblastoma who underwent resection after neoadjuvant chemotherapy.^8^ In pediatric patients with unresectable tumors, liver transplant (LT) combined with chemotherapy is the best option that provides long-term disease-free survival.^3^ A 10-year disease-free survival rate of 82% after LT has been reported in children with hepatoblastoma.^3^

Treatment of hepatoblastoma with liver resection (LR) or LT is associated with excellent long-term survival among pediatric patients.^9,10,11,12^ However, data on direct comparisons of LT with LR in pediatric patients with hepatoblastoma who were treated with chemotherapy are scarce. Moreover, the use of LT for pediatric patients with hepatoblastoma has not been examined in a modern cohort. We sought to examine national trends in the surgical treatment of pediatric patients with hepatoblastoma and to evaluate OS rates for pediatric patients with hepatoblastoma who received chemotherapy after undergoing LT or LR.

## Methods

### Data Source and Study Population

A registry-based retrospective cohort study was performed using data from the Surveillance, Epidemiology, and End Results (SEER) database for the years 2004 through 2016. The SEER database is supported by the Surveillance Research Program in the National Cancer Institute’s Division of Cancer Control and Population Sciences. The SEER program provides information on comprehensive demographic and cancer statistics covering 28% of the US population.^13^

In accordance with the Declaration of Helsinki,^14^ review by an institutional review board was not required for this study because the SEER database is publicly available without individually identifiable private information. All patient data are deidentified in the SEER database; thus, informed consent was also not required. This report follows the Strengthening the Reporting of Observational Studies in Epidemiology (STROBE) reporting guideline.^15^

Using the most recent release of the SEER database, we identified 583 patients younger than 18 years between January 1, 2004, and December 31, 2016. This calendar year interval was chosen to provide a sufficient information on tumor characteristics and chemotherapy use. Patients who did not receive a pathologic diagnosis (19 patients) and had not received chemotherapy (43 patients) were not included in the cohort. Cases were identified using a specific histologic code (*International Classification of Diseases for Oncology* code 8970).

Patients who did not undergo surgery for cancer were excluded (66 patients). Patients with unknown race/ethnicity (5 patients) and unknown tumor stage (7 patients) were also excluded. A total of 443 patients treated with chemotherapy and surgical therapy were selected for further analysis.

The following variables were retrieved from the SEER database: sex, age (categorized as age ≤1 year, ages 2-4 years, and ages 5-18 years), race/ethnicity (white, African American, Native American or Alaska Native, and Asian or Pacific Islander), year of diagnosis, tumor size, presence of multiple hepatic satellite lesions, disease extent, α-fetoprotein status, OS, and surgery type. The α-fetoprotein status was categorized as negative or within normal limits, positive or elevated, and other (ie, test not done, borderline, and unknown) in the SEER database.

### Statistical Analysis

Patient characteristics were compared using the χ^2^ test or Fisher exact test as appropriate. The Cuzick nonparametric test for trend was used to evaluate changes in treatment use over time. Surgery codes were described only after 1998 in SEER. To better describe the changes in the receipt of surgical therapy of patients with hepatoblastoma, we reviewed 617 pediatric patients with hepatoblastoma who underwent surgery from 1998 to 2016, as registered in the SEER database. Multivariable logistic regression analysis was used to examine factors independently associated with receipt of LT. Sex, age at diagnosis, race/ethnicity, year of diagnosis, tumor size, presence of multiple hepatic satellite lesions, disease extent, and α-fetoprotein status were included in the multivariable logistic regression analysis. The primary end point in this study was OS, which was defined as the time from diagnosis to death or date of last follow-up. Kaplan-Meier methods and the log-rank test were used for survival analysis. A Cox proportional hazards model was applied for multivariate survival analysis. All variables (sex, age at diagnosis, race/ethnicity, year of diagnosis, tumor size, presence of multiple hepatic satellite lesions, disease extent, α-fetoprotein status, and surgery type) were included in the multivariable Cox regression analysis. All tests of statistical significance were 2-sided, and statistical significance was established at *P* ≤ .05. Statistical analyses was performed with SPSS statistical software version 25.0 for Windows (SPSS). Data analysis was performed from April 18, 2019, to July 25, 2019.

## Results

We identified 583 patients treated with adjuvant chemotherapy and surgical therapy from January 1, 2004, through December 31, 2016, of whom 443 (mean [SD] age, 1.8 [2.6] years; 167 [37.7%] female) met the inclusion criteria (Table 1). Overall, 350 patients (79%) underwent LR, and 93 patients (21%) underwent LT. Solitary tumors (370 [84%]) were more common than multiple tumors (73 [16%]). Most patients received a diagnosis at age 1 year or younger (276 [62%]) or ages 2 to 4 years (129 [29%]); 38 patients (9%) received a diagnosis at ages 5 to 18 years. Local disease was present in 223 patients (50%).

**Table 1.** Characteristics of Pediatric Patients With Hepatoblastoma Who Underwent Liver Resection or Liver Transplant

Characteristic	Patients, No. (%) (N = 443)		*P* Value
	Liver Resection (n = 350)	Liver Transplant (n = 93)	
Sex			
Male	213 (61)	63 (68)	.22
Female	137 (39)	30 (32)	
Race/ethnicity			
White	269 (77)	69 (74)	.52
African American	28 (8)	8 (9)	
Native American or Alaska Native	11 (3)	1 (1)	
Asian or Pacific Islander	42 (12)	15 (16)	
Age at diagnosis, y			
≤1	224 (64)	52 (56)	.27
2-4	99 (28)	30 (32)	
5-18	27 (8)	11 (12)	
Year of diagnosis			
2004-2007	84 (24)	24 (26)	.89
2008-2011	110 (31)	27 (29)	
2012-2016	156 (45)	42 (45)	
α-Fetoprotein status			
Negative	8 (2)	1 (1)	.76
Positive	301 (86)	81 (87)	
Other^a^	41 (12)	11 (12)	
Tumor size, cm			
<5	37 (11)	8 (8)	.04
5-10	131 (37)	25 (27)	
>10	137 (39)	38 (41)	
Unknown	45 (13)	22 (24)	
Multiple hepatic satellite lesions			
No	306 (87)	64 (69)	<.001
Yes	44 (13)	29 (31)	
Disease extent			
Local	204 (58)	19 (20)	<.001
Regional	84 (24)	54 (58)	
Distant	62 (18)	20 (22)	
Overall survival			
Died	31 (9)	8 (9)	.92
Survived	319 (91)	85 (91)	

^a^ Not tested or borderline or unknown results.

Table 1 lists associations between variables and 2 surgical management strategies. Patients with multiple lesions were more likely to undergo LT than LR (31% vs 13%; *P* < .001). Patients with higher stage tumors were more likely to undergo LT than LR (local disease, 20% vs 58%; regional disease, 58% vs 24%; distant disease, 22% vs 18%; *P* < .001). A multivariable logistic regression model was used to examine factors independently associated with receipt of LT (Table 2). Patients with regional disease (odds ratio, 6.09; 95%, CI, 3.33-11.14; *P* < .001) or distant disease (odds ratio, 2.87; 95% CI, 1.37-6.01; *P* = .005) were more likely to undergo LT. The presence of multiple hepatic satellite lesions was associated with increased likelihood of the use of LT (odds ratio, 2.74; 95% CI, 1.47-5.09; *P* = .001).

**Table 2.** Multivariable Logistic Regression for the Receipt of Liver Transplant^a^

Characteristic	OR (95% CI)	*P* Value
Sex		
Male	1 [Reference]	
Female	0.67 (0.39-1.15)	.15
Race/ethnicity		
White	1 [Reference]	.86
African American	1.09 (0.42-2.80)	.86
Native American or Alaska Native	0.51 (0.06-4.41)	.54
Asian or Pacific Islander	1.24 (0.59-2.58)	.57
Age at diagnosis, y		
≤1	1 [Reference]	.90
2-4	1.14 (0.65-2.02)	.65
5-18	1.03 (0.43-2.50)	.95
Year of diagnosis		
2004-2007	1 [Reference]	.90
2008-2011	1.16 (0.58-2.30)	.68
2012-2016	1.14 (0.58-2.24)	.70
α-Fetoprotein status		
Negative	1 [Reference]	.75
Positive	2.14 (0.23-19.85)	.50
Other^b^	1.84 (0.18-18.97)	.61
Tumor size, cm		
<5	1 [Reference]	.10
5-10	1.06 (0.39-2.84)	.91
>10	1.39 (0.53-3.69)	.51
Unknown	2.72 (0.92-8.02)	.07
Multiple hepatic satellite lesions		
No	1 [Reference]	
Yes	2.74 (1.47-5.09)	.001
Disease extent		
Local	1 [Reference]	<.001
Regional	6.09 (3.33-11.14)	<.001
Distant	2.87 (1.37-6.01)	.005

Abbreviation: OR, odds ratio.

^a^ Binary logistic regression was performed. *P* ≤ .05 is regarded as statistically significant. If the OR is greater than 1, then receipt of liver transplant is more likely.

^b^ Not tested or borderline or unknown results.

**Figure 1.**
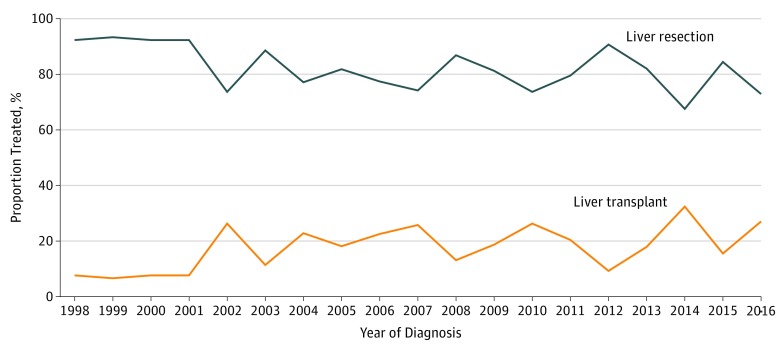
Trends in the Receipt of Liver Transplant and Liver Resection for Pediatric Patients With Hepatoblastoma From 1998 to 2016 Graph shows that there was a significant increase in the use of liver transplant over time, from 8% of patients in 1998 to 27% of patients in 2016.

Receipt of surgical therapy for pediatric patients with hepatoblastoma was examined from 1998 to 2016, and 766 pediatric patients with hepatoblastoma were identified. Of these, 617 had undergone surgical management. Overall, LR was more common (502 patients [81%]) than LT (115 patients [19%]). Figure 1 shows the proportional receipt of LT and LR from 1998 to 2016. There was a significant increase by 19% in the use of LT over time, from 1 of 13 patients (8%) in 1998 to 13 of 48 patients (27%) in 2016 (trend test, *P* = .02).

**Figure 2.**
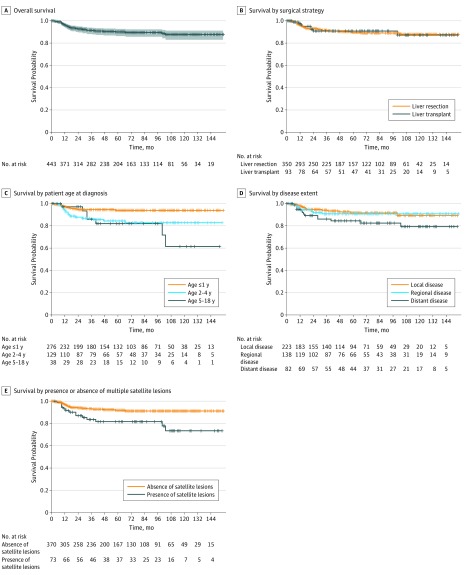
Kaplan-Meier Analysis of Pediatric Patients With Hepatoblastoma Graphs show Kaplan-Meier curves for overall survival (A), survival according to surgical management strategy (B), survival by patient age at diagnosis (C), survival by disease extent (D), and survival by the presence or absence of multiple hepatic satellite lesions (E). In panel A, shaded area indicates 95% CI.

The mean (SD) follow-up duration for the analyzed cohort (from 2004 to 2016) was 60 (44) months. The cumulative survival rates in our cohort were 90.0% (SE, 1.6%; 95% CI, 86.9%-93.2%) at 5 years and 87.7% (SE, 2.1%; 95% CI, 83.7%-91.8%) at 10 years (Figure 2A). Overall survival at 10 years was 87.2% (SE, 4.8%; 95% CI, 78.3%-97.1%) for patients undergoing LT and 87.8% (SE, 2.3%; 95% CI, 83.5%-92.4%) for those undergoing LR (*P* = .92) (Figure 2B). The 10-year OS rates were 93.8% (SE, 1.6%; 95% CI, 90.7%-97.1%) for patients who received a diagnosis at age 1 year or younger, 82.8% (SE, 3.8%; 95% CI, 75.6%-90.6%) for patients who received a diagnosis at ages 2 to 4 years, and 61.5% (SE, 13.7%; 95% CI, 39.7%-95.2%) for patients who received a diagnosis at ages 5 to 18 years (*P* = .002) (Figure 2C). The 10-year OS rates were 89.3% (SE, 3.1%; 95% CI, 83.4%-95.6%) for patients with local disease, 90.8% (SE, 2.7%; 95% CI, 85.7%-96.2%) for patients with regional disease, and 79.3% (SE, 5.5%; 95% CI, 69.3%-90.7%) for patients with distant disease (*P* = .08) (Figure 2D). Patients with single lesions experienced a 10-year OS of 91.2% (SE, 1.7%; 95% CI, 87.8%-94.6%) vs 73.4% (SE, 7.0%; 95% CI, 60.8%-88.6%) for those with multiple hepatic satellite lesions (*P* = .003) (Figure 2E).

Among the 276 patients who received a diagnosis at age 1 year or younger, the 10-year OS rates were 93.8% (95% CI, 89.3%-98.5%) for patients with local disease, 93.6% (95% CI, 88.3%-99.2%) for patients with regional disease, and 94.4% (95% CI, 87.2%-100%) for patients with distant disease (*P* = .99). Similarly, no significant difference in OS was found among patients who received a diagnosis at ages 2 to 18 years stratified by disease extent (eTable 1 in the Supplement). We then evaluated the association of surgery type with OS according to disease extent. No significant difference in OS was observed between the 2 surgical management strategies among patients stratified by disease extent (eTable 2 in the Supplement).

The use of LT was not associated with a survival benefit for patients treated with chemotherapy, compared with those undergoing LR (hazard ratio [HR], 0.716; 95% CI, 0.309-1.657; *P* = .44). Factors significantly associated with OS in the study population were age at diagnosis and the presence of multiple hepatic satellite lesions. Patients who received a diagnosis at ages 2 to 4 years were 128% more likely to die than those who received a diagnosis at age 1 year or younger (HR, 2.281; 95% CI, 1.111-4.684; *P* = .02). Patients who received a diagnosis at ages 5 to 18 years also had an increase in overall risk of death, compared with patients who received a diagnosis at age 1 year or younger (HR, 2.497; 95% CI, 0.943-6.608; *P* = .06). The presence of multiple hepatic satellite lesions was associated with increased overall risk of death (HR, 2.677; 95% CI, 1.293-5.543; *P* = .008) (Table 3). Patients with multiple hepatic satellite lesions were 168% more likely to die than those without multiple hepatic satellite lesions.

**Table 3.** Cox Proportional Hazards Regression Analysis for Overall Survival in Pediatric Patient With Hepatoblastoma

Characteristic	Overall Survival, HR (95% CI)	*P* Value
Sex		
Male	1 [Reference]	
Female	0.614 (0.299-1.260)	.18
Race/ethnicity		
White	1 [Reference]	.20
African American	2.549 (0.967-6.722)	.06
Native American or Alaska Native	1.341 (0.177-10.172)	.78
Asian or Pacific Islander	0.614 (0.183-2.054)	.43
Age at diagnosis, y		
≤1	1 [Reference]	.05
2-4	2.281 (1.111-4.684)	.02
5-18	2.497 (0.943-6.608)	.06
Year of diagnosis		
2004-2007	1 [Reference]	.42
2008-2011	0.729 (0.320-1.661)	.45
2012-2016	1.295 (0.560-2.994)	.55
α-Fetoprotein status		
Negative	1 [Reference]	.66
Positive	2.291 (0.265-19.783)	.45
Other^a^	1.680 (0.175-16.154)	.65
Tumor size, cm		
<5	1 [Reference]	.68
5-10	1.947 (0.430-8.819)	.39
>10	2.232 (0.507-9.819)	.29
Unknown	2.786 (0.506-15.343)	.24
Multiple hepatic satellite lesions		
No	1 [Reference]	
Yes	2.677 (1.293-5.543)	.008
Disease extent		
Local	1 [Reference]	.30
Regional	1.019 (0.437-2.375)	.96
Distant	1.798 (0.785-4.116)	.16
Surgery type		
Liver resection	1 [Reference]	
Liver transplant	0.716 (0.309-1.657)	.44

Abbreviation: HR, hazard ratio.

^a^ Not done or borderline or unknown.

## Discussion

In the present study, we analyzed SEER data to examine the use of LT and to evaluate the OS of pediatric patients with hepatoblastoma who were treated with chemotherapy after LT or LR. Our study revealed that pediatric patients with hepatoblastoma undergoing LT did not experience improved OS compared with those who underwent LR. There is a nationwide increase in the use of LT in the United States.^3,4^ Liver transplant was preferentially used in patients with regional or distant disease and those with multiple satellite lesions. To our knowledge, this is the largest report to date on the long-term survival outcomes for pediatric patients with hepatoblastoma treated with chemotherapy and surgical management.

Liver transplant plays an increasingly important role in the management of unresectable hepatoblastoma. According to a population-based study,^4^ 332 children underwent LT for unresectable hepatoblastoma from 1988 to 2010 in the United States. In 1998, 2 LTs were performed for hepatoblastoma.^4^ That number increased to 38 by 2010.^4^ Similarly, in our study, the use of LT increased significantly by 19%, from 8% (1 of 13 patients) in 1998 to 27% (13 of 48 patients) in 2016. After multivariable modeling, higher tumor stage and the presence of multiple hepatic satellite lesions were associated with greater odds of use of LT.

In the present study, among the 9 variables considered, age at diagnosis and multiple hepatic satellite lesions were shown to be independently associated with OS. We did not find any significant difference in survival between the 2 surgical management strategies for patients treated with chemotherapy. On multivariate cox regression analysis, the use of LT was not independently associated with OS (HR, 0.716; 95% CI, 0.309-1.657; *P* = .44), which was consistent with the findings of previous studies.^16,17^ McAteer et al^17^ analyzed data on patients with hepatoblastoma diagnosed between 1998 and 2009 and found that 5-year survival was 85.6% after LR and 86.5% after LT (*P* = .66). Ismail et al^16^ found that survival of children with hepatoblastoma was 71% for LR and 80% for LT (*P* = .82). We found a greater proportion of patients with regional and distant disease who underwent LT when compared with patients undergoing LR, perhaps explaining the nonsuperiority in OS among patients treated with LT.

Our results confirmed excellent 5-year OS for pediatric patients with hepatoblastoma treated with LR or LT, as reported elsewhere.^17,18,19,20^ Furthermore, the 5-year OS rate in our study was 90.0%, which was better than that of the aforementioned studies. This may be because we only included patients who received chemotherapy and surgical therapy in our study. In the present cohort study, patients who received a diagnosis at age 1 year or younger had better OS than those who received a diagnosis at ages 2 to 4 years, and the patients who received a diagnosis at ages 2 to 4 years had a 128% increased risk of death. Patients who received a diagnosis at ages 5 to 18 years also had increased overall risk of death, compared with patients who received a diagnosis at age 1 year or younger (HR, 2.497; 95% CI, 0.943-6.608; *P* = .06). In our subgroup analysis of patients who received a diagnosis at age 1 year or younger, there was no detectable association between tumor stage and OS. These findings are important because patients who received a diagnosis at age 1 year or younger represent the group who were most likely to benefit from chemotherapy administration and surgical management. In contrast to several other studies,^20,21,22^ tumor stage was not found to be independently associated with OS in the present study. However, this finding was consistent with previous studies describing hepatoblastoma as a chemotherapy-sensitive tumor.^3,23^ Thus, further large-scale comparative studies or large randomized clinical trials are needed to investigate the risk factors associated with inferior survival outcomes.

The management of pediatric patients with hepatoblastoma who presented with multiple satellite lesions at initial diagnosis is still challenging.^8,24,25^ Our analysis found that the presence of multiple hepatic satellite lesions was independently associated with long-term survival in pediatric patients treated with chemotherapy after LR or LT. Although patients with multiple hepatic satellite lesions were more likely to undergo LT, these patients were 168% more likely to die than those without multiple hepatic satellite lesions.

### Limitations

There are limitations to our study. First, although the data were derived from a large-scale tumor registration database, the study sample size was small because of the rarity of the disease. Second, the SEER database does not provide information about tumor recurrence or segmental involvement of the liver; thus, we were unable to include these factors into our analysis. Third, the follow-up time was short, because tumor information was missing before 2004. Fourth, information about comorbidities and underlying tumor biology or etiology is not available in the SEER database. Fifth, this study was a retrospective analysis with inherent selection bias.

## Conclusions

Liver transplant plays an important role in the surgical management of pediatric patients with hepatoblastoma who present with multifocal or metastatic disease. Our data suggest that there has been a significant increase in the use of LT for pediatric patients with hepatoblastoma. Among pediatric patients with hepatoblastoma receiving chemotherapy, LT was not associated with improved overall survival compared with LR. The ongoing challenge for pediatric surgeons treating pediatric patients with hepatoblastoma is to provide better long-term survival for each individual child after initial diagnosis with multifocal or metastatic hepatoblastoma.

## Notes

Supplement. **eTable 1.** Overall 5- and 10-Year Survival Rates After Chemotherapy and Surgical Management, According to Age at Diagnosis and Disease Extent **eTable 2.** Effect of Surgery Type on OS According to Disease Extent
