# Supplementary material for: Assessment of Survival of Pediatric Patients With Hepatoblastoma Who Received Chemotherapy Following Liver Transplant or Liver Resection
Source: JAMA Netw Open. 2019 Oct 4;2(10):e1912676. doi: 10.1001/jamanetworkopen.2019.12676 (PMC6784752; doi:10.1001/jamanetworkopen.2019.12676)
Supplement: Supplement. — eTable 1. Overall 5- and 10-Year Survival Rates After Chemotherapy and Surgical Management, According to Age at Diagnosis and Disease Extent eTable 2. Effect of Surgery Type on OS According to Disease Extent [file jamanetwopen-2-e1912676-s001.pdf]

Supplementary Online Content

Feng J, He Y, Wei L, et al. Assessment of survival of pediatric patients with hepatoblastoma who received chemotherapy following liver transplant or liver resection. *JAMA Netw Open*. 2019;2(10):e1912676. doi:10.1001/jamanetworkopen.2019.12676

eTable 1. Overall 5- and 10-Year Survival Rates After Chemotherapy and Surgical Management, According to Age at Diagnosis and Disease Extent
eTable 2. Effect of Surgery Type on OS According to Disease Extent

This supplementary material has been provided by the authors to give readers additional information about their work.

© 2019 Feng J et al. *JAMA Network Open.*

eTable 1. Overall 5- and 10-Year Survival Rates After Chemotherapy and Surgical Management, According to Age at Diagnosis and Disease Extent

Age at diagnosis	Disease extent	No. (%) of Patients	5-year OS (95% CI)	10-year OS (95% CI)	P
0-1 year	Local	149 (54)	93.8% (89.3%-98.5%)	93.8% (89.3%-98.5%)	0.99
	Regional	88 (32)	93.6% (88.3%-99.2%)	93.6% (88.3%-99.2%)	
	Distant	39 (14)	94.4% (87.2%-100%)	94.4% (87.2%-100%)	
2-18 years	Local	74 (44)	87.6% (79.3%-96.9%)	80.3% (65.9%-97.9%)	0.14
	Regional	50 (30)	85.2% (74.9%-97.0%)	85.2% (74.9%-97.0%)	
	Distant	43 (26)	75.2% (62.4%-90.8%)	67.2% (52.5%-86.0%)	

[a]Due to the limited number of patients with age 5-18 years, patients with age 2-4 years and 5-18 years were combined.

eTable 2. Effect of Surgery Type on OS According to Disease Extent

Disease extent	Surgery type	No. (%) of Patients	5-year OS (95%CI)	10-year OS (95%CI)	P
Local disease	Liver transplantation	19 (9)	100% (100%-100%)	75.0% (42.6%-100%)	0.88
	Liver resection	204 (91)	90.9% (86.4%-95.7%)	90.9% (86.4%-95.7%)	
Regional disease	Liver transplantation	54 (39)	89.3% (80.8%-98.7%)	89.3% (80.8%-98.7%)	0.72
	Liver resection	84 (61)	91.7% (85.5%-98.3%)	91.7% (85.5%-98.3%)	
Distant disease	Liver transplantation	20 (24)	88.2% (74.2%-100%)	88.2% (74.2%-100%)	0.47
	Liver resection	62 (76)	83.4% (74.1%-94.0%)	76.3% (64.1%-90.9%)	

© 2019 Feng J et al. *JAMA Network Open.*
